# ZYZ-168 alleviates cardiac fibrosis after myocardial infarction through inhibition of ERK1/2-dependent ROCK1 activation

**DOI:** 10.1038/srep43242

**Published:** 2017-03-07

**Authors:** Shanshan Luo, Tran Ba Hieu, Fenfen Ma, Ying Yu, Zhonglian Cao, Minjun Wang, Weijun Wu, Yicheng Mao, Peter Rose, Betty Yuen-Kwan Law, Yi Zhun Zhu

**Affiliations:** 1 Pharmacy and State Key Laboratory of Quality Research in Chinese Medicine, Macau University of Science and Technology, Macau, China; 2 Department of Pharmacology, School of Pharmacy, Fudan University, Shanghai, China; 3 Department of Pharmacy, Shanghai Pudong Hospital, Fudan University, Shanghai, China; 4 Department of Cardiology, Xin Hua Hospital, Shanghai Jiaotong University School of Medicine, Shanghai, China; 5 Instrumental Analysis Center, School of Pharmacy, Fudan University, Shanghai, China; 6 School of Biosciences, University of Nottingham, Loughborough, Leics LE12 5RD, UK.

## Abstract

Selective treatments for myocardial infarction (MI) induced cardiac fibrosis are lacking. In this study, we focus on the therapeutic potential of a synthetic cardio-protective agent named ZYZ-168 towards MI-induced cardiac fibrosis and try to reveal the underlying mechanism. ZYZ-168 was administered to rats with coronary artery ligation over a period of six weeks. Ecocardiography and Masson staining showed that ZYZ-168 substantially improved cardiac function and reduced interstitial fibrosis. The expression of α–smooth muscle actin (α-SMA) and Collagen I were reduced as was the activity of matrix metalloproteinase 9 (MMP-9). These were related with decreased phosphorylation of ERK1/2 and expression of Rho-associated coiled-coil containing protein kinase 1 (ROCK1). In cardiac fibroblasts stimulated with TGF-β1, phenotypic switches of cardiac fibroblasts to myofibroblasts were observed. Inhibition of ERK1/2 phosphorylation or knockdown of ROCK1 expectedly reduced TGF-β1 induced fibrotic responses. ZYZ-168 appeared to inhibit the fibrotic responses in a concentration dependent manner, in part via a decrease in ROCK 1 expression through inhibition of the phosphorylation status of ERK1/2. For inhibition of ERK1/2 phosphorylation with a specific inhibitor reduced the activation of ROCK1. Considering its anti-apoptosis activity in MI, ZYZ-168 may be a potential drug candidate for treatment of MI-induced cardiac fibrosis.

Myocardial infarction (MI) is a major cause of morbidity and mortality worldwide. It is estimated that in approximately 40% of patients, that survive the initial MI event, will undergo some cardiac remodeling that will later lead to heart failure^1^. Of specific interest is the aberrant remodeling that occurs in the left ventricle that is typically recognized as cardiac fibrosis. Cardiac fibrosis is a fatal cardiac disease characterized by cardiomyocytes dysfunction, abnormal differentiation of cardiac fibroblasts and interstitial fibrosis^2^. In the healthy heart, coordinated interactions between cardiomyocytes and cardiac fibroblasts are responsible for maintaining normal cardiac function. Indeed, cardiac fibroblasts are key source of the extracellular matrix (ECM) which provides a scaffold for cardiomyocytes^3^. However, in the ischemic heart, loss of cardiomyocytes alters this association leading to increased numbers of cardiac fibroblasts that can differentiate to form myofibroblasts^4^. In this instance, myofibroblasts end to express contractile proteins such as α–smooth muscle actin (α-SMA), moreover they appear to mount a healing response that involves increased expression and secretion of matrix metalloproteinase (MMPs) and collagen fibrillogenesis^5^. This uncontrolled activation of myofibroblasts eventually leads to the formation of cardiac fibrosis.

The MMP family plays a key role in the generation and propagation of fibrosis within cardiac tissues due to their ability to degrade the extra cellular matrix (ECM). Of the known members, MMP9 appears to play a major role in the cardiac remodeling process^6^. Clinical evidence has shown that plasma MMP9 levels correlate well with the severity of dilated cardiomyopathy after MI^7^,^8^, and that the levels of MMP9 are elevated immediately after MI, and remains high for at least two weeks^9^. In MMP9 deficient mouse models, functional loss of this enzyme contributes to reduced collagen deposition and attenuated ventricular dilation after MI^10^.

Similarly, Rho-associated coiled-coil containing protein kinases (ROCK) have also been implicated in cardiac remodeling since these proteins regulate cell shape by modulating actin filament dynamics^11^. Currently, two isoforms of ROCKs have been characterized and are designated as ROCK1 (also known as ROCK β or p160ROCK) and ROCK 2 (also known as ROCK α)^12^. In pulmonary fibrosis, ROCK activation drives cytoskeletal rearrangements that are responsible for the differentiation of fibroblasts to myofibroblasts^13^.

ROCK activation can be induced by multiple mechanical stimuli as well as several biochemical mediators, including TGF-β. For example, in pulmonary fibrosis, TGF-β drives ROCK 1 activation that initiates the formation of stress fibers during actin polymerization and promotes fibroblasts to myofibroblasts transition^14^. Thus, pharmacological targeting of ROCKs may provide a rational therapeutic target to reduce cardiac fibrosis. Indeed, pharmacologic inhibitors of ROCKs, such as Y27632 and Fasudil, have been shown to attenuate the development of vascular fibrosis and liver fibrosis induced by TGF-β^15^. Evidence also shows that in ROCK1^+/−^ haplo-insufficient mice decreased perivascular fibrosis is observed, suggesting that ROCK 1 signaling is important in the fibrotic response^16^. Moreover, in the heart, cardiac specific knockout of ROCK 1 has a protective effect against pressure overload-induced fibrosis^17^. Therefore, it would appear that ROCK 1 is an important mediator of TGF-β-induced fibrosis.

How ROCK 1 mediates these effects is only just coming to light with evidence suggesting that crosstalk with the MAPK signaling system may be involved. The MAPK pathway is activated by TGF-β in myofibroblasts and is a known downstream factor of TGF-β that mediates ROCK 1 expression in tissues^18^,^19^.

In the present study the role of the recently characterized cardioprotective agent 3,5-dimethoxy-4-(2-amino-3-prop-2-ynylsulfanyl-propionyl)-benzoic acid 4-guanidino-butyl ester (ZYZ-168, Supplementary Fig. 1) and its effects towards MI induced fibrosis were explored. ZYZ-168 is a novel conjugate of Leonurine (an alkaloid extracted from a traditional Chinese medicine called Leonurus)^20^ with S-Propargyl-L-cysteine (SPRC, also called as ZYZ-802)^21^. The protective effects of ZYZ-168 were tested using primary cultured cardiac fibroblasts in addition to a well-established animal model of myocardial infarction (MI). Cardiac function and fibrotic related proteins expression levels were determined as well as *in vitro* cell work to identify the molecular mechanisms that may be responsible for its putative effects.

## Results

### Mortality, heart weight and interstitial fibrosis

**Figure 1:**
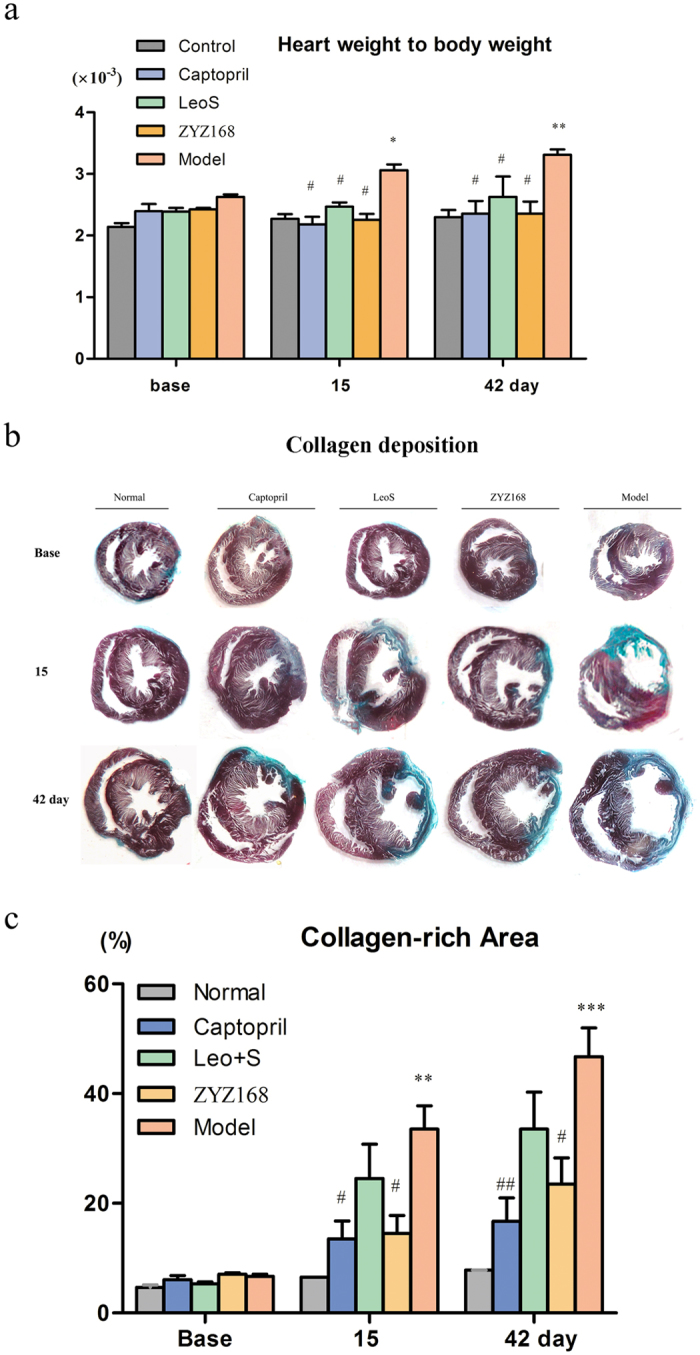
Ratio of left ventricle/body weight and fibrotic area determined by Masson trichrome staining. (**a**) Left ventricle/body weight ratio at 0 days, 15 days and 42 days of MI. Left ventricle wet weight increased in model group rats; ZYZ-168, LeoS and Cap treatment inhibited the increase of heart weight. (**b**) Representative images of Masson trichrome staining of hearts in different groups. (**c**) Fibrotic area was determined as the ratio of fibrotic scar (blue) average circumferences/left ventricle average inner circumferences. Data were expressed as mean ± SEM, six rats in each group were included. *P < 0.05 versus control group, **P < 0.01 versus control group, ***P < 0.001 versus control group, ^#^P < 0.05 versus model group, ^##^P < 0.01 versus model group.

A total of 80 rats were initially included in this study, including 12 rats in the control group. Seven rats died immediately due to post-myocardial infarction arrhythmias or hemathorax, five rats died one day after the surgery due to a large area of infarction. The left 56 rats were included in the following experiments. The dose of ZYZ-168 used in the current work was selected based on our previous studies^22^. Left ventricle/body weight (mg/g) ratios in different groups were comparable before the induction of MI. However, animals in model group showed an increased left ventricle/body ratio during two weeks of infarction, which is in accordance with report from another group^23^. Interestingly, left ventricle/body ratio was much smaller in ZYZ-168 treated group than in the model group during the initial two weeks of infarction, as shown in Fig. 1a. Fibrotic area was measured by Masson’s trichrome staining, as shown in Fig. 1b, the fibrotic area of the model group was increased at 15 days compared to that of control group. Figure 1c showed that ZYZ-168 treatment reduced the fibrotic area to 16 ± 4% at 15 days and 22 ± 5% at 42 days, respectively. Importantly, ZYZ-168 at 5 mg kg^−1^ day^−1^ showed a higher efficiency than that of leonurine combining with SPRC.

### ZYZ-168 improves cardiac function following long-term MI

**Figure 2:**
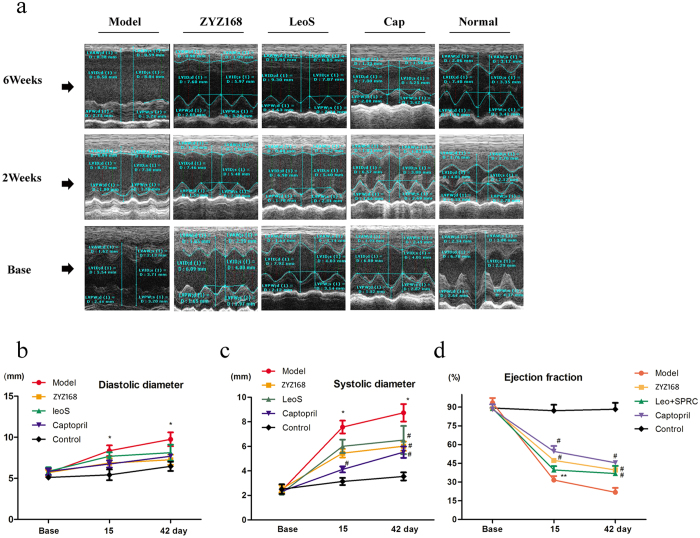
(**a**) Myocardial infarction caused abnormal expand of both cardiac systolic and diastolic volume, as well as reduction in ejection fraction over the 15 days and 42 days of infarction. (**b**) Statistical analysis of diastolic diameter, (**c**) systolic diameter and (**d**) ejection fraction after 15 days and 42 days infarction. ZYZ-168 treatment reduced systolic diameter and improved ejection function for 42 days treatment. ZYZ-168 treatment had no significant effect on diastolic diameter. n = 12 for each group. Data were analysed by one-way repeated measures ANOVA followed by post hoc analysis with Student–Newman–Keul’s test for multiple comparisons. *P < 0.05 versus control group, **P < 0.01 versus control group, ^#^P < 0.05 versus model group.

MI size was smaller in ZYZ-168 treated group than in the model group during the initial two weeks of infarction. Compared with the ZYZ-168 treated group, animals in the model group showed an enlarged left ventricle during six weeks of infarction, ZYZ-168 reduced infarction area and relieve the enlargement of left ventricle. Moreover, the mortality after MI was decreased with ZYZ-168 treatment as shown in Supplementary Fig. S2. Echocardiographic results of cardiac systolic diameter, diastolic diameter and ejection fraction were summarized in Fig. 2a–d. Echocardiographic basic values were comparable in each group. However, as shown in Fig. 2b, the LV end-diastolic diameter of the model group considerably increased at 15 days compared to that of control group. ZYZ-168 treatment failed to restore the diastolic diameter in this instance. In contrast, increased LV systolic diameter at 15 and 42 days of infarction in the model group was found and at these time points ZYZ-168 could effectively reduce LV systolic diameter (Fig. 2c). An index of cardiac function including ejection fraction was impaired in MI rats and this could be reversed by using ZYZ-168 for 15 day. ZYZ-168 treatment was enough to increase the ejection fraction by 22 ± 5% compared to the model group at day 42 (Fig. 2d).

Left Ventricular Posterior Wall (LVPW), Left Ventricular Internal dimension (LVID) and Left Ventricular Anterior Wall (LVAW) thickness are summarized in Supplementary Table S1. LVPWs or LVPWd thickness in rat of the model group was comparable to that of the control group. However, in MI rats reductions in LVAWd and LVAWs thickness (0.8 ± 0.3 mm vs. 2.1 ± 0.4 mm for control group, P < 0.01; 0.9 ± 0.2 mm vs. 3.5 ± 0.4 mm for control group, P < 0.001) at day 42 were observed. The echocardiographic results were consistent with the Masson trichrome staining, indicating that the dilated left ventricle observed in MI rats is a risk factor for heart rupture. Animals treated with ZYZ-168 had a greater LVAW thickness both in systolic and diastolic phases, which could potentially reduce the risk of heart rupture.

### ZYZ-168 reduces remodeling in the rat myocardium

**Figure 3:**
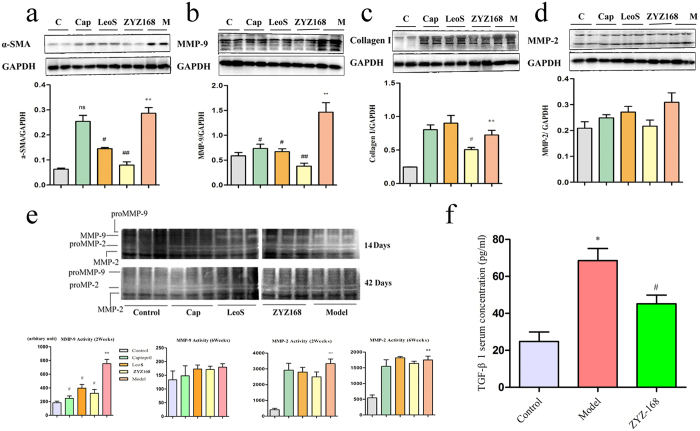
Expression of α-SMA, Collagen I, MMP9 in peri-infarct tissue over 42 days of infarction and the activities of MMP2 and MMP-9 after 15-day and 42-day infarction. Long-termed infarction significantly enhanced (**a**) α-SMA, P = 0.008, (**b**) Collagen I, P = 0.004 and (**c**) MMP9, P = 0.0078 expression in peri-infarct tissue, ZYZ-168 treatment attenuated increase of these proteins. (**e**) MMP-9 was expressed at the 92-kD band (pro-form) and the 84-kD band (active form); MMP-2 was evident at the 72-kD (pro-form) and the 62-kD band (active form). MMP9 activity increased obviously at 15 days of infarction compared with that of control group, and ZYZ-168 treatment inhibited MMP9 activity at the initial of 15 days. MMP2 activity was unchanged over the experiment period except that in control group, in which MMP9 activity was undetectable. n = 6 for each group (**f**) ZYZ-168 reduced serum TGF-β1 at 15 days of infarction (n = 8 for each group). Data were expressed as means ± SEM, *P < 0.05 versus Control group, **P < 0.01 versus control group, ^#^P < 0.05 versus model group, ^##^P < 0.01 versus model group.

The pathogenesis of tissue remodeling in the heart corresponds with the increased expression of α-SMA in the myocardium^24^, and changes to the rates of synthesis and degradation of the extracellular matrix. It is now widely accepted that increased deposition of ECM is accompanied with the activation and secretion of MMP9 and Collagen I^25^. In the current study the expressions of α-SMA, Collagen I and MMP9 in the peri-infarct region of heart tissues after 42 days following MI was determined. As shown in Fig. 3a–c, western blot analysis revealed key changes in the expression of α-SMA, Collagen I, and MMP9. In this instance, all three fibrotic related proteins showed higher expression rates in the model (MI) group as compared with the controls. Furthermore, in animals treated with ZYZ-168, a considerably reduction in α-SMA, collagen I and MMP9 were found. MMP9 is not typically expressed in healthy heart tissues but is known to be induced following MI induction. In contrast, MMP2, a constitutively expressed protein, remained unchanged in the peri-infarct region (Fig. 3d,e) and is in accordance with previous studies^26^. Interestingly, MMP-9 activation was not found in the control group or in the captopril treated group, but was detected in the model, ZYZ-168 and LeoS treated groups after two weeks of MI. MMP9 activity was higher in the model (MI) group, compared with that in the control groups during the two weeks following MI induction, but was much lower at six weeks post MI. ZYZ-168 treatment inhibited MMP9 activity at two weeks but showed no effects at six weeks of MI. Both pro-MMP2 and MMP2 were observed in all groups, except for the control group, and no changes were observed in the expression levels or activities of this protein. It has been reported that direct activation of MMP-2 typically occurs within 2–3 minutes of reperfusion following ischemia^27^, and this may explain the undetectable changes in MMP2 activity found in this work. We further detected the level of TGFβ1 in serum of myocardial infarction rats. Figure 3f demonstrated that compared with control rats, rats with 2 weeks of myocardial infarction showed higher serum TGFβ1 level, and ZYZ-168 treatment reduced the level.

### TGF-β-induced fibroblast to myofibroblasts conversion was dose-dependently inhibited by ZYZ-168

**Figure 4:**
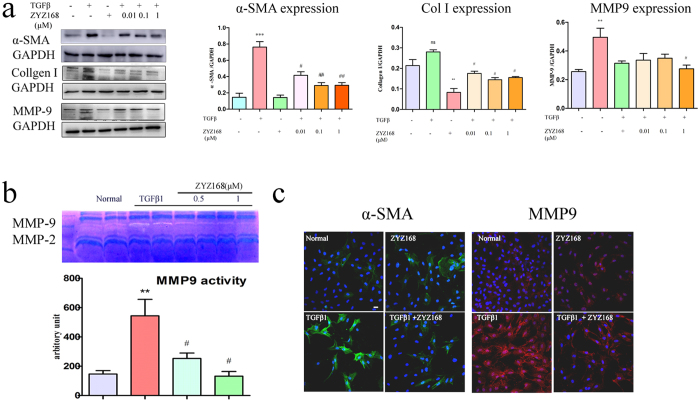
Levels of α-SMA, Collagen I, MMP9 in TGF-β stimulated cardiac fibroblasts. (**a**) 48 hrs of TGF-β stimulation increased fibrotic related α-SMA, Collagen I and MMP9 expression in cardiac fibroblasts, ZYZ-168 pretreatment dose-dependently reduced the expression of these fibrotic related proteins. Values were means ± SEM of five independent tests. (**b**) MMP9 and MMP2 activity was determined by gelatin zymography. MMP9 was evident at 84-kD band (active form) and MMP2 was evident at 62-kD band (active form). Cardiac fibroblasts exposed to TGF-β for 48 hrs showed enhanced MMP9 activity rather than MMP2 activity. ZYZ-168 treatment dose-dependently inhibited MMP9 activity. Values were means ± SEM of five independent tests. (**c**) Representative immunofluorescent results for expression of α-SMA and MMP9 in TGF-β-stimulated cardiac fibroblasts. α-SMA: Green, MMP9: Red, magnification: 200×. *P < 0.05 versus Normal group, **P < 0.01 versus Normal group, ^#^P < 0.05 versus TGF-β-treated group, ^##^P < 0.01 versus TGF-β-treated group.

Previous studies have shown that TGF-β stimulation of fibroblast promotes myofibroblasts conversion and stimulates ECM formation^28^. Activation of endogenous TGF-β is observed during the development of cardiac fibrosis in response to MI. To determine the effects of ZYZ-168 on myofibroblasts differentiation, cardiac fibroblasts were isolated from the healthy left ventricular tissues of adult animals and stimulated with 500 nM TGF-β. Fibroblast to myofibroblasts differentiation was evident as determined by the increased expression of α-SMA in cells and due to the formation of α-SMA–positive stress fibers, as shown in Fig. 4a and c. ZYZ-168 treatment concentration dependently inhibited α-SMA formation in cardiac fibroblasts exposed to TGF-β. Moreover, 1 μM ZYZ-168 pretreatment was also found to reduce TGF-β induced MMP9 activity (Fig. 4b). Fluorescent staining results in Fig. 4c supported this result and showed that pretreatment with ZYZ-168 prevented TGF-β-induced secretion of MMP9 by myofibroblasts. Similarly, it is now widely recognized that Collagen I is a major component of the ECM and that abnormal deposition of Collagen I is observed in fibrotic diseases. As shown in Fig. 4a, ZYZ-168 treatment decreased expression of TGF-β-induced Collagen I in a concentration dependent manner.

### ZYZ-168 alleviated TGF-β-induced MMP9 secretion through inhibiting ERK1/2 phosphorylation

**Figure 5:**
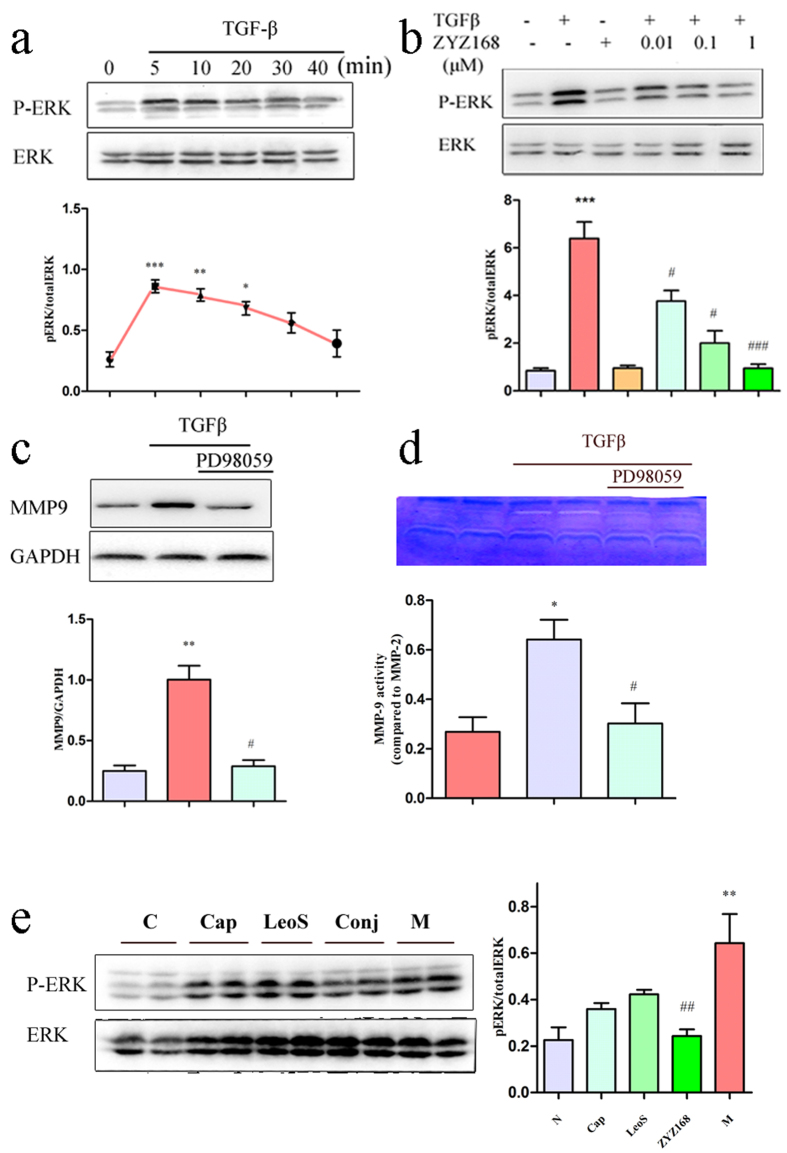
Exposure of cardiac fibroblasts to 500 nM TGF-β led to a time-dependent (**a**) phosphorylation of ERK1/2 (p-ERK1/2) and (**b**) ZYZ-168 treatment dose-dependently rescued level of phosphor-ERK1/2. (**c**) Cardiac fibroblasts were pretreated with 10 μM PD98059 (PD) before TGF-β stimulation. PD98059 could inhibit MMP9 expression and (**d**) reduced MMP9 activity. (**e**) Phosphor-ERK1/2 expression in peri-infarct tissue was determined by western blotting, it enhanced in model group (M), and ZYZ-168 treatment reduced its level. *In vitro* data were means ± SEM of five independent samples. *In vivo* data were means ± SEM of six independent samples. (**a**–**d**): *P < 0.05, **P < 0.01 and ***P < 0.001 versus non-treated group, ^#^P < 0.05 and ^##^P < 0.01 versus TGF-β-treated group. (**e**) **P < 0.01 versus control group, ^##^P < 0.01 versus model group.

In order to illustrate the underlying mechanism of the anti-fibrotic effects of ZYZ-168, we initially focused on the cardinal TGFβ/Smad2/3 pathway, we did observe the activation of Smad2 in cardiac fibroblasts subjected to TGFβ stimulation, unfortunately, ZYZ-168 pre-treatment failed to inhibit the activation of Smad2 in this condition, as shown in Supplementary Fig. S3a and b. On the other hand, more and more evidences have shown that non-cardinal pathways in TGF-β signaling are activated in the process of fibrosis. In TGF-β-mediated epithelial to mesenchymal transition (ETM), for example, activation of ERK1/2 pathway is indispensible for fibroblast-like cell formation^29^. To determine the role of the MAPK pathway on TGF-β signaling, cardiac fibroblasts were treated with TGF-β and the phosphorylation status of ERK1/2, JNK and p38 were determined. Upon TGF-β treatment, the phosphorylation of MAPKs was increased as expected, as shown in Fig. 5a and Supplementary Fig. S3. A concentration dependent reduction in the phosphorylation status of ERK1/2 by ZYZ-168 was found in TGF-β stimulated cardiac fibroblasts (Fig. 5b). ZYZ-168 had no effect on the phosphorylation status of JNK or p38 (See Supplementary Fig. S3). Interestingly, the expression and activity of MMP9 were also reduced using the ERK1/2 inhibitor PD98059. Indeed, as shown in Fig. 5c and d, PD98059 treatment led to a substantial decrease in MMP9 expression and its activity in cardiac fibroblasts treated with TGF-β. This finding suggested that ERK1/2 signaling was involved in MMP9 expression in cardiac fibroblasts. To confirm this observation and to determine whether ZYZ-168 could inhibit ERK1/2 phosphorylation status *in vivo*, western blotting was performed. Figure 5e showed that administration of ZYZ-168 decreased ERK1/2 phosphorylation in MI rats. In contrast, SDS-PAGE results in Supplementary Fig. S4 showed that the phosphorylation status of JNK and p38 were not altered by ZYZ-168. These results demonstrated that ZYZ-168 inhibited TGF-β-induced MMP9 activation via the inhibition of ERK1/2 phosphorylation rather than that of JNK or p38.

### ZYZ-168 alleviated TGF-β-induced MMP9 secretion through inhibiting ROCK 1 expression

**Figure 6:**
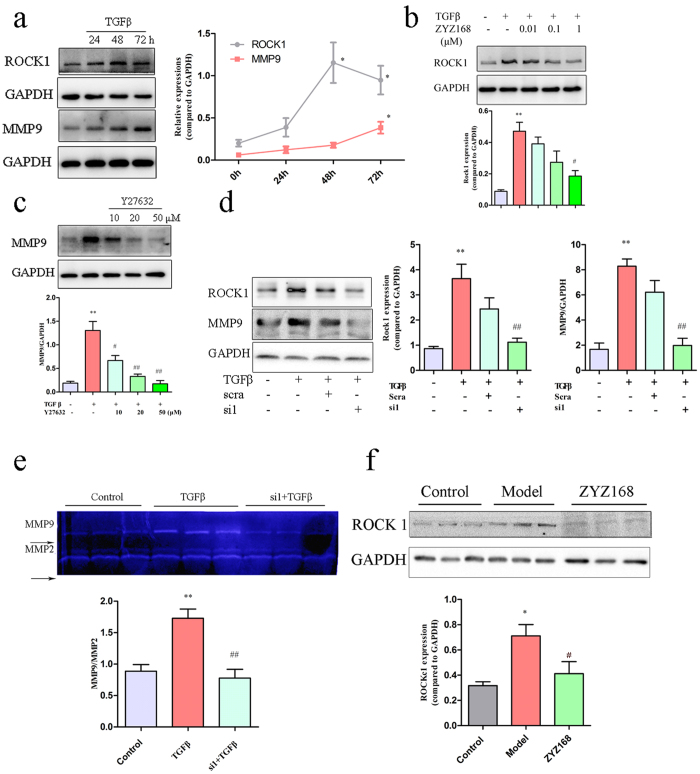
Exposure of cardiac fibroblasts to 500 nM TGF-β led to a time-dependent (**a**) increase of ROCK1 expression and (**b**) ZYZ-168 dose-dependently reduced ROCK1 expression. (**c**) Cardiac fibroblasts were pretreated with 10 μM, 20 μM and 50 μM Y27632 for 24 hrs, followed by stimulated with TGF-β for 48 hrs. Y27632 dose-dependently reduced MMP9 expression. (**d**) Cardiac fibroblasts were transfected with ROCK1 siRNA1 (si1) or scramble siRNA (scra) for 48 hrs prior tostimulated with TGF-β, expression of ROCK1 and MMP9 (**e**) and activity of MMP9 were determined. (**f**) ROCK1 expression in peri-infarct tissue was determined by western blotting, ROCK1 expression enhanced in model group, and ZYZ-168 treatment reduced ROCK1 level. Data *in vivo* were represented as means ± SEM, and three independent samples were adopted. *In vitro* data were means ± SEM of five independent experiments. *In vivo* data were means ± SEM of three independent samples. *P < 0.05, **P < 0.01 versus non-treated group or control group, ^#^P < 0.05, ^##^P < 0.01 versus TGF-β-treated group or model group.

Except for the MAP kinase pathways, the non-cardinal pathways responding for TGF-β stimulation also include Rho-like GTPase signaling pathways. To date, many studies on TGF-βsignaling pathway have focused on activation of RhoA/ROCK 1^23^,^30^, we next determined whether this pathway was a target of ZYZ-168. Cardiac fibroblasts were treated with TGF-β for 24 hrs, 48 hrs, and 72 hrs respectively and the expression of ROCK 1 and MMP9 at each time point were determined. Within 48 hrs and 72 hrs post TGF-β treatment, significantly higher levels of ROCK 1 (1.17 ± 0.61, 48 hrs, P = 0.0227; 0.91 ± 0.47, 72 hrs, P = 0.047) were observed as compared to the control cells (0.21 ± 0.09; see Fig. 6a). Similarly, MMP-9 levels were also elevated following TGF-β treatment and showed a higher level at 72hrs (TGF-β treatment, 0.42 ± 0.11 vs. control group, 0.07 ± 0.01, Fig. 6a). Statistical analysis showed that the expression level of MMP9 was strongly correlated with that of ROCK 1 (Fig. 6a). In contrast, a significant decrease (P = 0.035) in the expression of ROCK1 in ZYZ-168 pretreated cardiac fibroblasts stimulated with TGF-β was found. Indeed, as shown in Fig. 6b, pretreatment with ZYZ-168 reduced the expression level of ROCK 1 to near control cell levels. These results suggested that ZYZ-168 may reduce TGF-β-induced MMP9 expression via the ROCK1 pathway.

To support this observation, it was important to determine whether ROCK 1 inhibition was sufficient to reduce MMP9 levels. Using the commercially available Rho kinase inhibitor Y27632, cardiac fibroblasts were treated with the respective inhibitor prior to stimulation with TGF-β. Y27632 at concentrations of 10–50 μM showed dose dependent inhibition of the expression of MMP9. Indeed, compared to the TGF-β-treated cells, the expression of MMP9 was reduced by almost 80% in cells treated with 50 μM Y27632, as shown in Fig. 6c. Since Y27632 has been found to inhibit additional kinases^31^,^32^, siRNA mediated knockdown of ROCK1 was also performed. Four siRNAs targeting rat ROCK 1 gene were synthesized and transfected into cardiac fibroblasts. 48 hours following transfection, ROCK 1 and MMP9 protein expression were determined. All four siRNA inhibitors had comparable efficiency and for easy of study ROCK1 siRNA1 was used in further experiments (Supplementary Fig. S5). ROCK 1 knockdown resulted in a 50% reduction in MMP9 expression as compared with that in the TGF-β-treated cells, shown in Fig. 6d,e, this result indicated that ROCK 1 had a functional role in regulating MMP9 expression in cardiac cells. *In vivo* confirmation was used to show that long-term myocardial infarction resulted in enhanced ROCK 1 expression in the peri-infarct heart. Moreover, Fig. 6f showed that ZYZ-168 could decrease MI-induced ROCK 1 activation in tissue of dangerous area. These data provided compelling evidence that ROCK1 participates in TGF-β-induced MMP9 activation.

### TGF-β mediated activation of ROCK1 is ERK1/2 dependent

**Figure 7:**
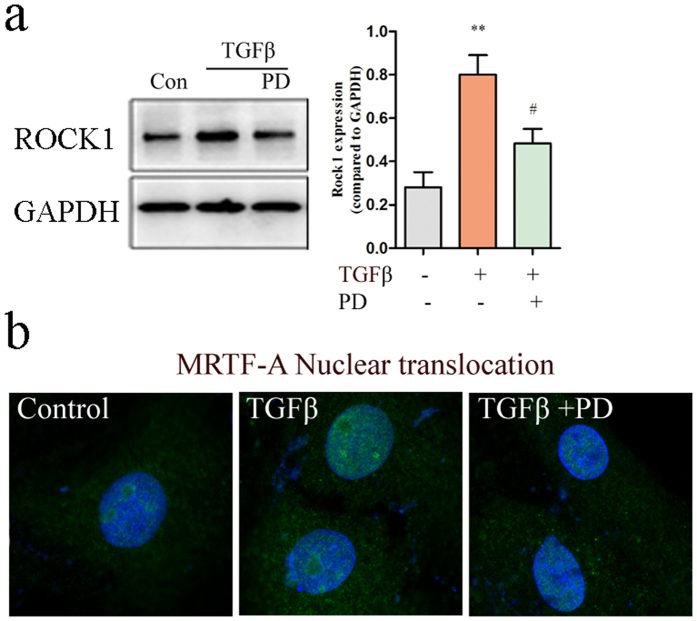
Cardiac fibroblasts were pretreated with PD98059 (10 μM), followed by incubation with TGF-β, MRTF-A nuclear translocation and ROCK1 expression were determined. (**a**) Inhibition of ERK1/2 phosphorylation effectively inhibited ROCK1 expression induced by TGF-β. Data represent means ± SEM of five independent experiments. **P < 0.01 versus non-treated or Control (Con) group, ^#^P < 0.05 versus only TGF-β-treated group. (**b**) Immunofluorescent staining showed that incubation with TGF-β enhanced the nuclear translocation of MRTF-A (Green). And PD98059 seemed to inhibit MRTF-A nuclear translocation.

In this study it was found that ERK1/2 phosphorylation potentiated the expression of ROCK 1 in cardiac fibroblasts exposed to TGF-β. Inhibition of ERK1/2 phosphorylation using PD98059 reduced ERK1/2 phosphorylation (Supplementary Fig. S6a) and effectively prevented TGF-β induced ROCK1 expression (Fig. 7a), while inhibition of ROCK 1 activity using Y27632 had no effects on ERK1/2 phosphorylation status (See Supplementary Fig. S6b). Moreover, TGF-β-induced myocardin-related transcription factor-A (MRTF-A) nuclear translocation was altered by ERK1/2 inhibition. Typically, MRTF-A is localized in the cytoplasm by binding to G actin, and is activated following ROCK1 mediated actin polymerization. These events lead to the liberation of MRTF-A from G actin and expose a nuclear localization sequence (NLS) of MRTF-A. The NLS leads to increased nuclear import of MRTF-A and accumulation of MRTF-A in the nucleus^33^. As expected, Fig. 7b demonstrated that PD98059 treatment reduced TGF-β-induced MRTF-A nuclear translocation. These data suggested that ERK1/2 acts as a key upstream regulator of the ROCK 1 signaling pathway.

**Figure 8:**
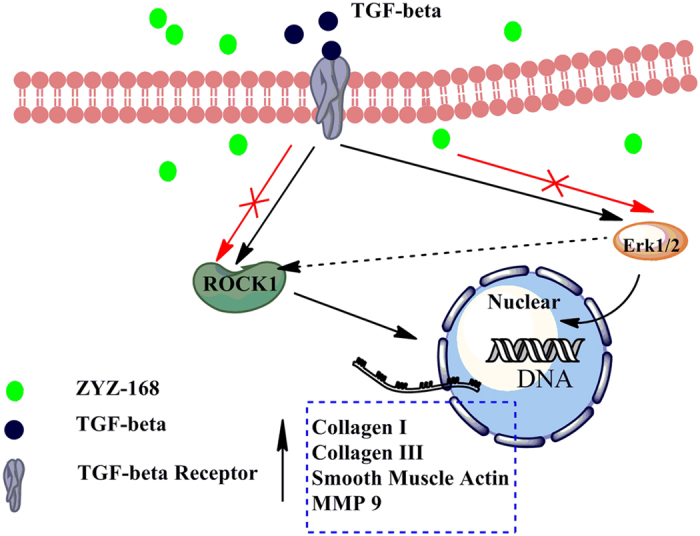
Activation of ROCK1 and ERK1/2 branches of the TGF-β1 pathway took part in the differenciation of cardiac fibroblasts to myofibroblasts. In cardiac fibroblasts, TGF-β1 can phosphorylate ERK1/2 and activate ROCK 1, phosphorylated ERK1/2 can also increase expression of ROCK1. ZYZ-168 treatment inhibited TGF-β1-induced ERK1/2 phosphorylaton, resulting in hypoactivity of ROCK1. Black arrows indicate TGF-β1-induced myofibroblasts differenciation; red arrows indicate effects of ZYZ-168 treatment; dashed lines indicate the assumed mechanism which might be involved in ZYZ-168’s anti-fibrotic effects.

Collectively, as shown in Fig. 8, our data highlights that activation of ROCK1 and ERK1/2 take part in the differenciation of cardiac fibroblasts to myofibroblasts stimulated by TGF-β1, and ERK1/2 at least in part influences ROCK1 expression. ZYZ-168 reduced TGF-β-induced cardiac fibroblasts transition through inhibiting phosphorylaton of ERK1/2 and activation of ROCK1.

## Discussion

Cardiac fibrosis is a major health problem worldwide, patients surviving from ischemia develop cardiac fibrosis that is characterized by scar formation, and tissue sclerosis contributing to a impaired heart function^24^. Therefore, the identification of anti-fibrotic agents that can be utilized in the development of future therapies will be invaluable in treating this disease. Despite this, it would appear that the availability of clinical drugs that can be used to treat cardiac fibrosis is limited. Therefore, the current study was performed to determine whether ZYZ-168 had any therapeutic potential in reducing cardiac fibrosis and to identify the molecular mechanisms responsible.

Results from this work indicate that changes in fibrosis occur primarily within the first 14 days, and that ZYZ-168 prevents the development of fibrotic lesions during the very early stages of disease progression. In previously work ZYZ-168 was shown to specifically reduce cardiomyocyte cell death in the acute phase of MI^22^. This finding may well explain the smaller fibrotic lesion formed in ZYZ-168 treated rats found in this study. At 42 days post MI induction, changes in the ventricular geometry in rats was apparent with the enlargement of the left ventricular corresponding to impaired cardiac function and increased risk of cardiac rupture. In animals treated with ZYZ-168, reduction in markers of cardiac damage and fibrosis development were found. For example, a reduction in eccentric hypotrophy-related parameters like Masson staining, ejection fraction index and thickness of left ventricular anterior wall, were noted in ZYZ-168 treated animals (See Figs 1 and 2 and Supplementary Table S1).

How ZYZ-168 mediates these effects became the next focus of this piece of work^34^. Myofibroblasts are known to secret collagens (such as collagen I, III, IV) that mediate synthesis and repair of the ECM that contributes to the development of scar tissue. Similarly, this cell type also secretes MMP9 that plays a pivotal role in this remodeling process. Indeed, previous studies have correlated the fibrotic response with a time-dependent increase in MMP9 activity^9^. And in multiple models of tissue injury, early activation of MMPs occurs prior to the end stage-fibrotic process^35^. In this work, MMP9 activity reached maximal activity levels during the first 14 days and declined thereafter; MMP9 was hardly detectable at 42 days of MI. On the other hand, the expression of α-SMA and deposition of extracellular collagen was found to increase more steadily and displayed an increase at 42-day post MI. The simultaneous increase in tissue collagens coupled with the appearance of α-SMA, as described in the present study, supports the cardiac fibroblasts to myofibroblasts conversion and changes to ECM deposition. Daily ZYZ-168 treatment substantially reduced both the activation of MMP-9 the expression of α-SMA as shown in Fig. 3 and in Fig. 4. These results support the anti-fibrosis potential of ZYZ-168 in long-term MI.

How ZYZ-168 influences ECM remodeling was the next focus of attention. Currently, a number of signaling systems have been reported to be involved in cardiac fibrosis progression, these including the classic SMAD3/4 downstream factor, TGF-β mediated fibrosis occurs via a non-canonical pathway that likely involves receptor tyrosine kinases (RTKs), MAPK signaling^36^ along with PI3K^37^. Previous work in our lab has shown that the alkaloid leonurine effectively reduced ERK1/2 phosphorylation in stimulated microglial cells^38^. This discovery prompted us to consider whether ZYZ-168 can inhibit TGF-β-induced ERK1/2 phosphorylation during fibrosis. Consistent with our previous work, TGF-β stimulation of cardiac fibroblast induced the phosphorylation status of ERK1/2. As shown in Fig. 5, ERK1/2 phosphorylation was inhibited by ZYZ-168.

Interestingly, several studies have shown that TGF-β can induce the Rho GTPase signaling pathway that triggers the downstream effector protein ROCK1 in pulmonary and renal fibrosis^39^. ROCK 1 activated by TGF-β mediates changes in cellular actin organization via multiple downstream targets, including the serum response factor/myocardin-related transcription factor A (SRF/MRTF-A) complex^40^. Because there is no specific method for direct ROCK1 activity detection, the nuclear translocation of its downstream effectors (such as MRTF-A) is applied to indicate activity of ROCK1. We demonstrate that an increase in ROCK1 expression in long-term MI rats and in cardiac fibroblasts exposed to TGF-β and that this could be inhibited by ZYZ-168. Interestingly, MMP9 seems like a target gene of ROCK 1 signaling pathway, since the up regulation of MMP9 correlated with the enhanced expression of ROCK 1. To date, multiple target genes of ROCK1 pathway are known to be key drivers of fibrosis^41^,^42^,^43^, and it is not unreasonable to suggest that MMP9 may be one of these.

At present, in cells stimulated by TGF-β, both ERK1/2-MAPK and RhoA-ROCK pathways can be activated with both being known to control different cellular processes^44^. However, few studies have explored any potential cross talk between each of these signaling systems. A few studies have shown that ROCK 1 deficiency can impair MAPK signaling, however, little is known regarding the effects of ERK1/2-MAPK deficiency on the ROCK 1 signaling pathway^18^,^45^. The current work indicates that upon exposure to TGF-β, ERK1/2 phosphorylation in cardiac fibroblasts increases within 30 mins, and that the application of the ERK1/2 phosphorylation inhibitor PD98059 inhibits ROCK 1 and MRTF-A nuclear translocation. These results suggest that ERK1/2 might be an upstream mediator of ROCK1. Based on this above findings it is proposed that the ability of ZYZ-168 to reduce cardiac fibrosis appears to correspond with its ability to reduce ERK1/2 phosphorylation, that prevents ROCK 1 activation and the expression of fibrotic proteins.

In conclusion, the current study demonstrates that ZYZ-168 effectively reduces cardiac fibrosis and improves cardiac function in long-term myocardial infarction rats. These cardioprotective properties appear to be due to changes to MAPK and RhoA/ROCK1 signaling pathway. Importantly, ZYZ-168 attenuates TGF-β induced MMP9 expression, inhibits collagen synthesis *in vitro*, and prevents the conversion of cardiac fibroblasts to myofibroblasts. Interestingly, the co-administration of purified leonurine combined with SPRC was also found to preserve cardiac function, however, this drug combination failed to display any obvious anti-fibrotic potential (See Figs 1b and 3a). These results are consistent with our previous research using an acute myocardial infarction (AMI) model^22^. Indeed, in such work it was found that ZYZ-168 (a leonurine SPRC drug conjugate) exerted higher efficacy than that of leonurine combined with SPRC. Together with its anti-apoptosis potential, ZYZ-168 may be a promising drug candidate for the treatment of myocardial infarction induced cardiac fibrosis.

## Methods

### Animals

Male Sprague-Dawley rats weighing 200–250 g were kept in an animal room under SPF conditions (temperature 24 ± 1 °C; humidity 55–60%; 12 h/12 h light/dark cycle) with free access to food and water for one week before the experiment. All animals received human care in accordance with the Guide for the Care and Use of Laboratory Animals, published by the National Institutes of Health (NIH publication no. 85-23, revised 1996). The investigation was approved by Institutional Animal Care and Use Committee of Fudan University and thus performed in accordance with the ethical standards laid down in the 1964 Declaration of Helsinki and its later amendments. The results of all studies involving animals are reported in accordance with the ARRIVE guidelines^46^.

### Surgical preparation of animals

Rats were anaesthetized with an i.p. injection of pentobarbital sodium (40 mg·kg^−1^). If re-dosing was required during surgery, 5 mg·kg^−1^ pentobarbital sodium was given. Adequacy of anaesthesia was monitored by pedal response. The rats were mechanically ventilated with oxygen-enriched room air by a rodent respirator ventilated (Ugo, Comerio, Italy)via tracheal intubation (at 60·min^−1^ frequency). A thoracotomy was performed at the fourth intercostal space, hearts were “popped out” from chest and the left anterior descending artery (LAD) was permanently ligated 1–2 mm distal from the left auricle and arterial cone with a 7–0 polypropylene suture under sterile conditions. Control animals (12 rats) underwent the same procedure except that the LAD was left untied. During the surgery, body temperature was maintained constant at 37 °C by a heating pad. Rats survived from MI were randomly assigned into four groups: Captopril (Cap, 20 mg·kg^−1^·day^−1^), Leonurine + SPRC (LeoS, 7.5 + 3.5 mg·kg^−1^·day^−1^), ZYZ-168 (5 mg·kg^−1^·day^−1^) and Model, with 14 rats in each group. Drug administration was started 24 hours after surgery and was continued for 6 weeks. Rats in the control group and in MI group were injected with the same volume of saline.

### Echocardiographic Measurement

At the time points of before surgery, two weeks and six weeks after the surgery, echocardiography was conducted. Rats were anaesthetized with an i.p. injection of pentobarbital sodium (40 mg·kg^−1^) and fixed in supine position on a heating pad. Heart rate was monitored with a standard limb lead II electrocardiogram (ECG) and maintained at 50 ± 5 per minute during the echocardiography. Cardiac functions were evaluated by transthoracic echocardiography with an ultrasound machine (Visual Sonics Inc., Toronto, Canada) with a 716 probe. Left ventricular systolic diameter (LVSD), Left ventricular diastolic diameter (LVDD), ejection fraction (EF) and fractional shortening (FS) were derived automatically by the High-Resolution Electrocardiograph System. Left ventricular internal dimension in systole and diastole (LVIDs, LVIDd); left ventricular anterior wall in systole and diastole (LVAWs, LVAWd); left ventricular posterior wall in systole and diastole (LVPWs, LVPWd) were obtained from the parasternal long axis with an M-mode test. All measurements were averaged over three consecutive cardiac cycles.

### Histological analysis of collagen deposition

At the time points of two weeks and six weeks after the surgery, rats were anaesthetized with an *i.p*. injection of 1% (g/mL) pentobarbital sodium (40 mg·kg^−1^). After rats were adequate anaesthesia, thoracotomy was conducted and hearts were collected. Ratio of heart weight (wet) to body weight was determined. For histological staining, hearts were sliced along the short-axis plane at the level of one third of the distance from the atrioventricular ring to the apex. The hearts were then fixed in 4% paraformaldehyde, embedded with paraffin, and cross-sectionally cut into 5 mm thick sections along the centre of the fibrotic scar. The left part of the heart was quickly frozen by liquid nitrogen for detection of protein expressions. Masson’s trichrome staining was applied to evaluate collagen deposition. Cardiac fibrosis was assessed by calculating the ratio of fibrotic scar (blue) average circumferences to left ventricular average inner circumferences. All quantitative evaluations were carried out by ImagePro Plus software (version 6.0, Media Cybernetics, Bethesda, MD, USA).

### Measurement of Serum TGF-β1

TGF-β1 concentrations were measured by a commercially available enzyme-linked immuno sorbent assay (ELISA) kit (Boatman Biotech CO. Ltd, Shanghai, China). All analytical steps were performed according to the manufacturer’s recommended procedures. And concentrations are presented as mean of eight samples.

### Isolation and culture of cardiac fibroblasts

Cardiac fibroblasts were isolated as previously described^47^. Rats weighting 200–250 g were anaesthetized with an i.p. injection of pentobarbital sodium (40 mg·kg^−1^). Briefly, Left ventricular was isolated and minced into small chunks (less than 1mm^3^). The tissue was then placed into 5 ml of 0. 125% trypsin and was digested at 37 °C for 10 minutes. The supernatant was collected and centrifuged at 1000 × g for 3 minutes to obtain cell pellet and the harvested cells were re-suspended in DMEM supplemented with 1% (v/v) penicillin and streptomycin and 10% (v/v) fetal bovine serum. The process was repeated for 6–7 times to further disaggregate the cells. Cardiac fibroblasts were purified by removal of unattached cardiomyocytes. Cardiac fibroblasts at the second passage were used in the experiments.

### *In vitro* drug treatment

Cardiac fibroblasts were pretreated with various concentrations of ZYZ168 (0.01, 0.1 and 1 μM respectively) or equal volume of DMSO for 4 h, followed by stimulated with 500 nM TGF-β (ABclonal, CA, USA) for 48 hours. Cells were stimulated with TGF-β for different times to determine the MRTF-A nuclear translocation, activation of MAPKs and expression of ROCK 1. Phosphorylation of EKR1/2, JNK and p38-MAPKs were inhibited by pretreatment cells with PD98059 (10 μM), SP600125 (10 μM), and SB 202190 (10 μM) respectively. ROCK 1 activity was inhibited by incubating cells with Y27632 for 24 hours. All the inhibitors were purchased from Selleck Chemicals LLC.

### Protein isolation and western blotting analysis

Protein was extracted from the cultured cardiac fibroblasts and the peri-infarct tissue of rats. The protein concentration was determined by BCA protein assay kit (Beyotime, Nantong, China). Protein samples were subjected to 8% SDS-PAGE and blotted to nitrocellulose. The blots were blocked by 5% non-fat milk, followed by probed with antibodies against α-SMA (Abcam, Cambridge, UK); Collagen I (Merck, Kenilworth, NJ, USA); MMP9 and ROCK1 (BioLegend, San Diego, CA, USA); ERK1/2, phospho-ERK1/2, JNK, phospho-JNK, p38-MAPK, phospho-p38-MAPK and GAPDH (Cell Signaling Technology, Irvine, CA, USA) overnight at 4 °C. Membranes were then washed with TBST and incubated with secondary antibody (SantaCruz Biotechnology, Santa Cruz, CA, USA) for 2 h at room temperature. Membranes were washed three times with TBST and ECL western blotting detection reagent (Millipore) was used to develop the immunoblots. Protein bands were visualized by ChemiDoc™ MP Imaging System (Bio-Rad, Hercules, CA, USA).

### Detection activity of MMPs by gelatin zymography

Gelatin zymography was performed as described previously. In brief, freshly isolated peri-infarct tissue were homogenized in lysis buffer, the supernatant was collected and mixed with Tris-Glycine SDS Sample Buffer (2×). Samples (50 μg) were directly loaded onto standard polyacrylamide gels containing 1% (g/mL) gelatin (Sigma-Aldrich Corp. St. Louis, MO USA). After electrophoresis, the gels were washed twice in rinsing buffer containing 2.5% Triton X-100 for 1 h to remove SDS. Then they were incubated in developing buffer (50 mM Tris·HCl, pH 7.6, 5 mM CaCl2, 1 μM ZnCl_2_, 0.2 mM NaCl, and 0.02% (w/v) Brij-35) for 48 hours at 37 °C. After incubation, the gels were stained with 0.125% Coomassie blue R-250 for 2 hours followed by destained with a solution containing 10% (v/v) acetic acid and 20% (v/v) methanol. The gels were dried until clear bands of gelatinolysis appeared on a dark blue background. Zymographic activity was quantified using a digital image analyser (ImageJ, NIH).

### Transfection of ROCK1 siRNA

ROCK1 siRNA1, siRNA2, siRNA3 and Negative Control siRNA were synthesized by GeneChem Inc. (GeneChem, Shanghai, China). The Cardiac fibroblasts were transfected with siRNAs against ROCK1 by using Lipo2000 (Invitrogen, Carlsbad, CA, USA) according to the manufacturer’s instructions. Briefly, the siRNAs (50 nM) and Lipo2000 (3 μL) were each diluted to 100 μL with Opti-MEM medium and then mixed. The siRNA-lipid complex was incubated for 20–25 minutes at room temperature. After incubation, the complex was directly added into 6-well plate with just-confluent cells. Six hours after the transfection, the medium was discarded and replaced with fresh medium and the cells were incubated for 24 h before stimulated with TGF-β. To determine the knockdown efficiency, each of the three siRNA duplexes (50 nM) and a combination of these three siRNA duplexes in equal ratios (named “siRNA4”) were used and 48 hours after transfection, the expression of ROCK1 and MMP-9 were determined. For determination of MMP9 enzymatic activity in ROCK1 deficient cells, the supernatant was collected 48 hours after the transfection and applied for gelatin zymography.

### Immunofluorescent staining for α-SMA, MMP9 and MRTF-A

Cardiac fibroblasts were seeded on a confocal dish and fixed with 4% formaldehyde. Cells were then immersed in 0.3% Triton X-100 for 15 min and mounted by blocking solution (10% BSA and 0.1% NaN3) for 1 h at room temperature. After incubation with rabbit polyclonal anti-α-SMA (1:1000), anti-MMP9 (1:200) and anti-MRTF-A (1:500) antibodies overnight at 4 °C, cells were washed with 0.01 M PBS and subsequently incubated with Alexa Fluor 488-conjugated goat anti-rabbit or Alexa Fluor 594-conjugated goat anti-rabbit secondary antibody (1:1000, Molecular Probes, Eugene, OR, USA). Cells were observed by using a Carl Zeiss LSM710 confocal microscopy (Carl Zeiss, Inc., Jena, Germany). For each group, three independent tests were required and micrographs of 3–5 fields were taken for each well. All micrographs were taken under the same conditions to make sure the fluorescent intense reflecting the content of the protein.

### Statistical analysis

Statistical analyses were performed by GraphPad Prism 5 (La Jolla, CA, USA). Data were presented as mean ± SEM. Because the sample size was small (less than 10), D’Agostino-Pearson omnibus test was optioned for normality analysis and data that passed normality test (α > 0.05) was used for the following analysis. Echocardiographic data were analyzed by one-way repeated measures ANOVA followed by post hoc analysis with Student–Newman–Keul’s test for multiple comparisons, other data such as differences in the intensity of α-SMA, collagen I, MMP9 and the percentage of collagen-rich area were analyzed by ANOVA followed by Bonferroni’s post hoc test. P values less than 0.05 were considered to be statistically significant. All the artworks were created by Adobe PhotoShop CS6.

## Additional Information

**How to cite this article**: Luo, S. *et al*. ZYZ-168 alleviates cardiac fibrosis after myocardial infarction through inhibition of ERK1/2-dependent ROCK1 activation. *Sci. Rep.* **7**, 43242; doi: 10.1038/srep43242 (2017).

**Publisher's note:** Springer Nature remains neutral with regard to jurisdictional claims in published maps and institutional affiliations.

## Supplementary Material

Supplementary Materials
