# Supplementary material for: ZYZ-168 alleviates cardiac fibrosis after myocardial infarction through inhibition of ERK1/2-dependent ROCK1 activation
Source: Sci Rep. 2017 Mar 7;7:43242. doi: 10.1038/srep43242 (PMC5339863; doi:10.1038/srep43242)
Supplement: Supplementary Materials [file srep43242-s1.pdf]

ZYZ-168 alleviates cardiac fibrosis after myocardial infarction through inhibition of ERK1/2-dependent ROCK1 activation

Shanshan Luo [1, 2], Tran Ba Hieu[2] , Fenfen Ma [4], Ying Yu[2, 3],Zhonglian Cao [5], Minjun Wang [1, 2], Weijun Wu[2], Yicheng Mao[2], Peter Rose [6], Betty Yuen-Kwan Law[1] and Yi Zhun Zhu [1,2*]

Supplementary Fig.S1: Structure of ZYZ-168

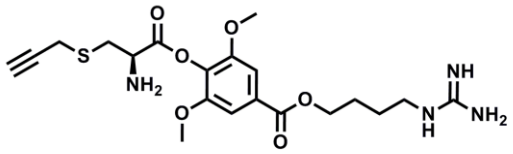

Supplementary Fig.S1: Structure of ZYZ-168

Supplementary Fig.S2: ZYZ-168 reduced infarction size and improved survival rate after myocardial infarction

a **b**

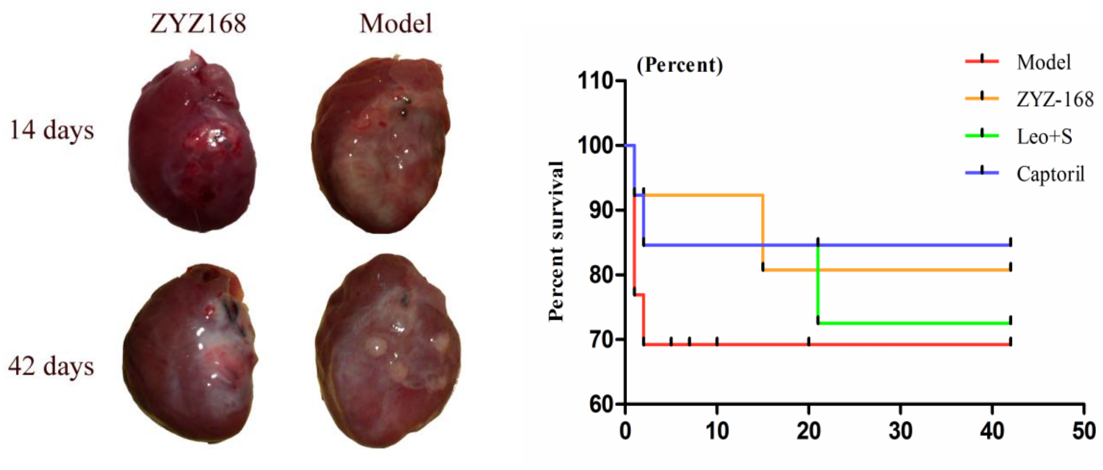

Supplementary Fig.S2 a) ZYZ-168 reduced infarct size and inhibited enlargement of left ventricle. Hearts from ZYZ-168-treated group and model group were isolated and photographed. Model group showed lager area of infarction after 14 days of MI.

Moreover, after 42 days of MI, left ventricle were significantly enlarged and showed dialated cardiomyopathy. ZYZ-168 treatment reduced left ventricular remodeling. b) ZYZ-168 improved the survival rate of rats after myocardial infarction.

Supplementary Fig.S3: ZYZ-168 did not reduce Smad2/3, P38 or JNK phosphorylation in cardiac fibroblasts exposed to TGF-β

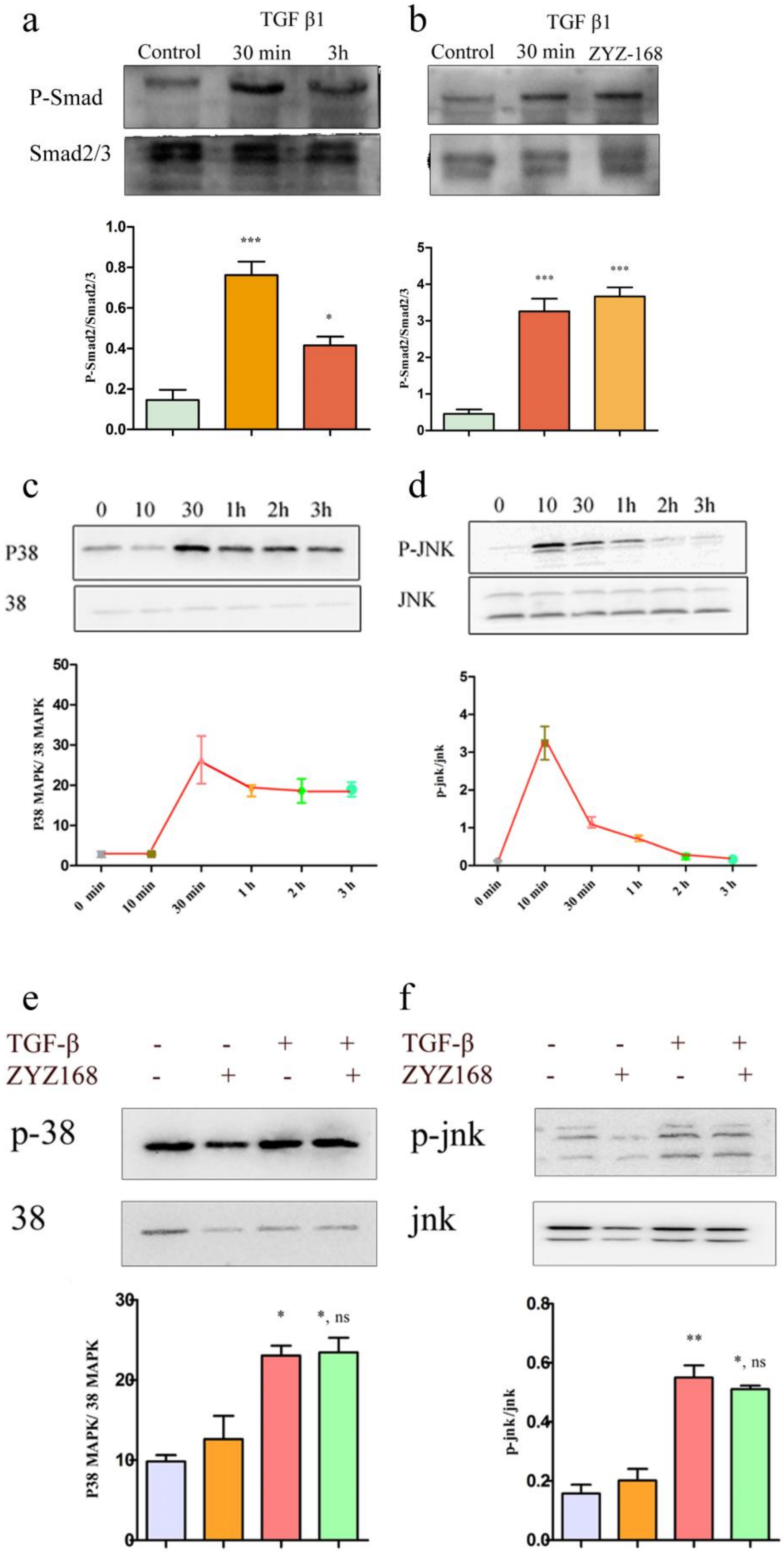
a
TGF β1
Control
30 min
3h
P-Smad
Smad2/3
P-Smad2/Smad2/3
b
TGF β1
Control
30 min
ZYZ-168
P-Smad2/Smad2/3
c
0
10
30
1h
2h
3h
P38
38
P38 MAPK/ 38 MAPK
0 min
10 min
30 min
1 h
2 h
3 h
d
0
10
30
1h
2h
3h
P-JNK
JNK
p-jnk/jnk
e
TGF-β
ZYZ168
p-38
38
P38 MAPK/ 38 MAPK
*, ns
f
TGF-β
ZYZ168
p-jnk
jnk
p-jnk/jnk
*, ns

Supplementary Fig.S3 Cardiac fibroblasts were stimulated with TGF-β for different time points, and phosphorylation of Smad2, JNK and p-38 were determined. TGF-β induced (a) and (c) significant increase of Smad2, p-38 phosphorylation at 30 min and (d) JNK phosphorylation at 10 min. (b), (e) and (f) Pre-treatment with ZYZ-168 for 4 hours had no effects on phosphorylation of Smad2, p38 or JNK. Data were means ±SEM of three independent experiments. *P<0.05 versus non-treated cells, **P<0.01 versus non-treated cells, ***P<0.005 versus non-treated cells.

Supplementary Fig.S4: ZYZ-168 did not reduce P38 or JNK phosphorylation in peri-infarct heart.

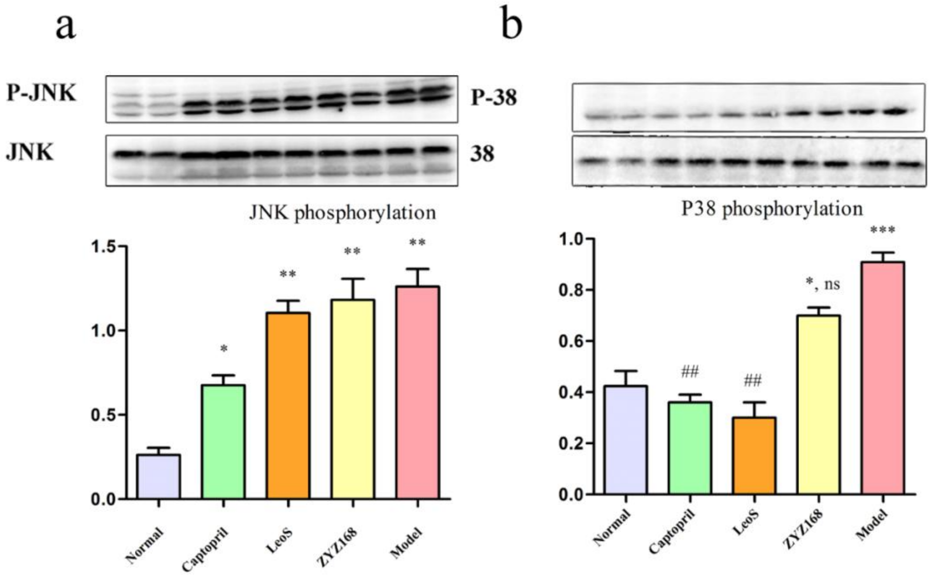

Supplementary Fig.S4 Phosphorylation of JNK and p-38 in peri-infarct tissue was determined by Western blotting. Phosphorylation of JNK (a) and p-38 (b) enhanced significantly in Model group, but ZYZ-168 treatment could not reduce their phosphorylation levels. Data were means±SEM of six independent samples. *P< 0.05, **P< 0.01, ***P< 0.001 versus Control group, $^{\#\#}$P<0.05 versus Model group.

Supplementary Fig.S5: ROCK1 siRNA1-4 showed comparable efficacy, MMP9 expression was also inhibited with ROCK1 knockdown.

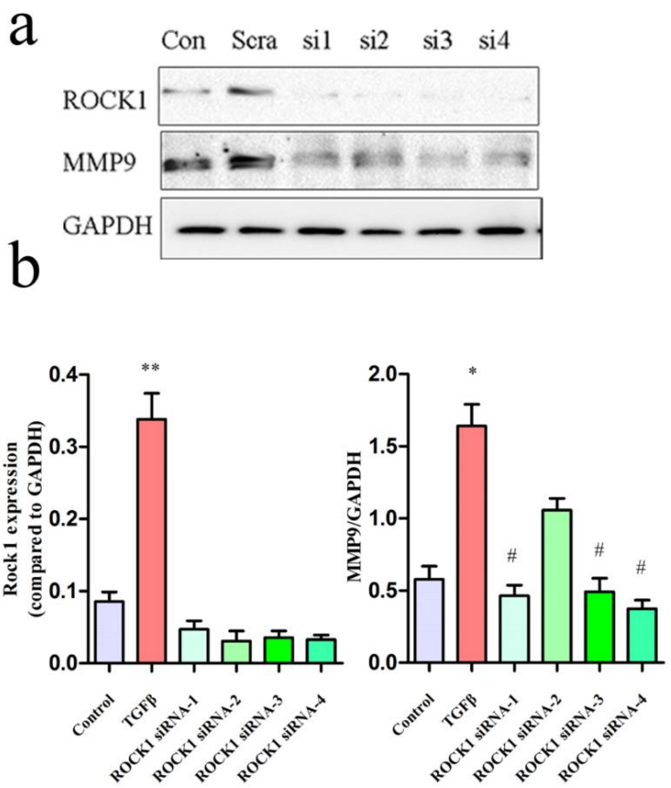

Supplementary Fig.S5 Cardiac fibroblasts were infected with Scramble siRNA (Scra) and ROCK1 siRNAs 1-4. ROCK1 expression was determined, and expression of MMP9 in different groups was also determined. (a) Representative western blotting results of ROCK1 and MMP9. (b) Statistical analysis of the expression of ROCK1 and MMP9. Data were means ±SEM of three independent experiments. $^{*}P<0.05$, $^{**}P<0.01$ versus control cells, $^{\#}P<0.01$ versus scramble siRNA transfected cells.

Supplementary Fig.S6: ROCK1 inhibition failed to alleviate TGFβ-induced phosphorylation of ERK1/2.

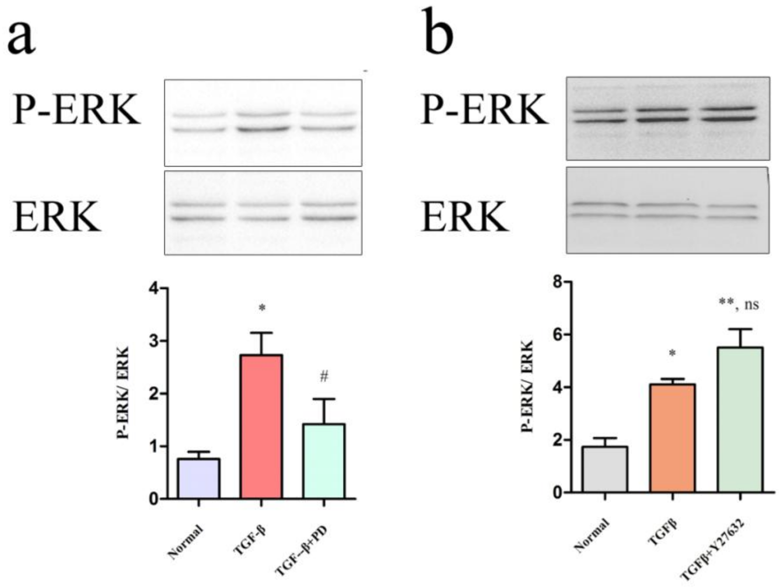

Supplementary Fig.S6 (a) Cardiac fibroblasts were incubated with PD98059 and TGFβ for 24 hours, the phosphorylation of ERK1/2 was determined after incubation. PD98059 significantly reduced phosphorylation of ERK1/2 induced by TGF β. (b) Cardiac fibroblasts was pretreated with Y27632 to inhibit activity of ROCK1 for 24 hours, followed by stimulated with TGF β for another 30 min, and phosphorylation of ERK1/2 was determined. Inhibition of ROCK1 activity showed no effects on ERK1/2 phosphorylation in TGFβ treated cardiac fibroblasts. Data were means ±SEM of three independent experiment. *P<0.05, **P<0.01 versus Normal cells, $^{\#}$P<0.01 versus cells exposed to TGFβ.

Supplementary Table. S1: The summarization of the indexes of cardiac functions by echocardiography.

	Sham			Cap			LeoS		
Index (mm)	Base	14	42day	Base	14	42day	Base	14	42day
LVPW;d	1.6±0.4	1.5±0.2	2.1±0.1	1.5±0.2	1.6±0.5	2.0±0.4	1.4±0.2	1.4±0.5	2.0±0.1
LVPW;s	2.3±0.2	2.6±0.2	3.7±0.3	2.3±0.1	2.6±0.3	3.2±0.5	2.4±0.1	2.0±0.7	2.8±0.2
LVID;d	4.8±0.2	5.6±0.3	6.9±0.9	5.7±0.5	6.7±0.6	6.6±1.0	5.3±0.5	7.9±1.2	8.4±1.1
LVID;s	2.9±0.3	3.0±0.5	2.7±0.6	2.8±0.4	4.8±0.5#	4.2±0.3#	2.8±0.4	6.5±1.1	6.7±1.0
LVAW;d	1.6±0.2	1.4±0.2	2.1±0.4	1.8±0.2	1.2±0.2	1.5±0.2#	1.9±0.2	0.8±0.2	1.1±0.3
LVAW;s	2.0±0.1	2.3±0.1	3.5±0.4	2.4±0.3	1.5±0.2	2.0±0.3#	2.0±0.3	0.9±0.2	1.4±0.2

	Conj			Model		
Index (mm)	Base	14	42day	Base	14	42day
LVPW;d	1.4±0.4	1.8±0.1	2.0±0.2	1.6±0.2	1.5±0.2	2.1±0.5
LVPW;s	2.4±0.1	2.7±0.5	3.0±0.6	2.4±0.3	1.7±0.2	2.5±0.5
LVID;d	5.8±0.2	6.7±0.2	7.0±0.5	5.3±0.6	8.4±0.2	9.7±0.9*
LVID;s	3.3±0.3	4.3±0.6#	5.3±0.5#	3.3±0.2	7.5±0.2*	9.0±0.7*
LVAW;d	2.0±0.1	1.3±0.4#	1.5±0.3#	2.0±0.3	0.8±0.1*	0.8±0.3*
LVAW;s	2.5±0.1	2.1±0.3#	2.0±0.4#	2.4±0.1	0.9±0.2*	0.9±0.2*

Supplementary Table. S1: LVPWd and LVPWs, Left ventricular posterior wall in diastole or systole; LVIDd and LVIDs, left ventricular internal dimension in diastole of systole; LVAWd and LVAWs, left ventricular anterior wall in diastole or systole. n=12 for each group. Data are expressed as mean± SEM, *P< 0.5 versus Control group, **P< 0.1 versus Control group, #P<0.5 versus Model group.
